# smORFunction: a tool for predicting functions of small open reading frames and microproteins

**DOI:** 10.1186/s12859-020-03805-x

**Published:** 2020-10-14

**Authors:** Xiangwen Ji, Chunmei Cui, Qinghua Cui

**Affiliations:** Department of Biomedical Informatics, Department of Physiology and Pathophysiology, Center for Noncoding RNA Medicine, School of Basic Medical Sciences, Peking University, 38 Xueyuan Rd, Beijing, 100191 China

**Keywords:** Small open reading frame, Microprotein, Function prediction, Gene expression

## Abstract

**Background:**

Small open reading frame (smORF) is open reading frame with a length of less than 100 codons. Microproteins, translated from smORFs, have been found to participate in a variety of biological processes such as muscle formation and contraction, cell proliferation, and immune activation. Although previous studies have collected and annotated a large abundance of smORFs, functions of the vast majority of smORFs are still unknown. It is thus increasingly important to develop computational methods to annotate the functions of these smORFs.

**Results:**

In this study, we collected 617,462 unique smORFs from three studies. The expression of smORF RNAs was estimated by reannotated microarray probes. Using a speed-optimized correlation algorism, the functions of smORFs were predicted by their correlated genes with known functional annotations. After applying our method to 5 known microproteins from literatures, our method successfully predicted their functions. Further validation from the UniProt database showed that at least one function of 202 out of 270 microproteins was predicted.

**Conclusions:**

We developed a method, smORFunction, to provide function predictions of smORFs/microproteins in at most 265 models generated from 173 datasets, including 48 tissues/cells, 82 diseases (and normal). The tool can be available at https://www.cuilab.cn/smorfunction.

## Background

With the deeper understanding of human genome, GENCODE [1], FANTOM [2] and other projects have annotated a large number of coding and/or non-coding genes. It is known that human genome has ~ 20,000 protein-coding RNAs and potentially more non-coding RNAs [1, 3]. However, by matching initiation and termination codons, millions of potential open reading frames (ORFs) can be identified, which is far more than the number of functional elements currently discovered [4]. Among them, the ORF with a length of less than 100 codons is defined as a small ORF (smORF), and the protein translated by smORF is called microprotein [5]. By the advantages of ribosome profiling sequencing (Ribo-seq), researchers can identify ribosome-binding RNA fragments, which are RNAs in translation, providing strong evidence to support the annotation of smORFs [6]. sORFs.org [7] and small proteins database (SmProt) [8] collected over 2 million and 160,000 human smORFs respectively. Another study used de novo transcript assembly to improve annotation accuracy, and identified over 7,500 smORFs [9].

Mass spectrometry (MS) enables the certification of the existence of microprotein [10]. Several studies have demonstrated the role of microproteins in humans and other mammals. For example, dwarf open reading frame (DWORF), a 34 amino acids (aa) microprotein, enhances muscle contraction by increasing the calcium uptake of sarcoplasmic reticulum [11]. Microprotein inducer of fusion (Minion), specifically expressed during skeletal muscle development and regeneration, is found to induce cell fusion and muscle formation [12]. In addition, microproteins also play regulatory roles in proliferation [13, 14], cell respiration [15, 16], and immune regulation [17].

Although the functions of a few microproteins have been studied, the functions of the majority of smORFs remain unknown. Therefore, it is emergently necessary to develop computational tools to predict the functions of microproteins. ProteomeHD measured the co-regulatory relationships of proteins by MS and then predicted the functions of proteins and microproteins [18]. Functional smORF-encoded peptides predictor (FSPP) used MS, Ribo-seq and RNA sequencing (RNAseq), predicted the function of microproteins by co-expression and co-location networks [19]. However, quantification of large number of microproteins or their RNAs is difficult due to the small sizes and large number. For example, ProteomeHD covered ~ 10,000 proteins, a small fraction of which were microproteins, much smaller than the potential number of microproteins, while FSPP used only 38 samples. Here, we propose a computational method, smORFunction, to predict the function of 526,443 smORFs/microproteins in at most 265 models generated from 173 datasets, including 48 tissues/cells, 82 diseases (and normal). Then we confirmed that smORFunction can successfully predict the function of microproteins by case studies and database validations. Moreover, we developed a web tool of our method, providing potential helps for the studies of smORFs and microproteins.

## Results

### smORF RNA quantification based on microarray

**Fig. 1:**
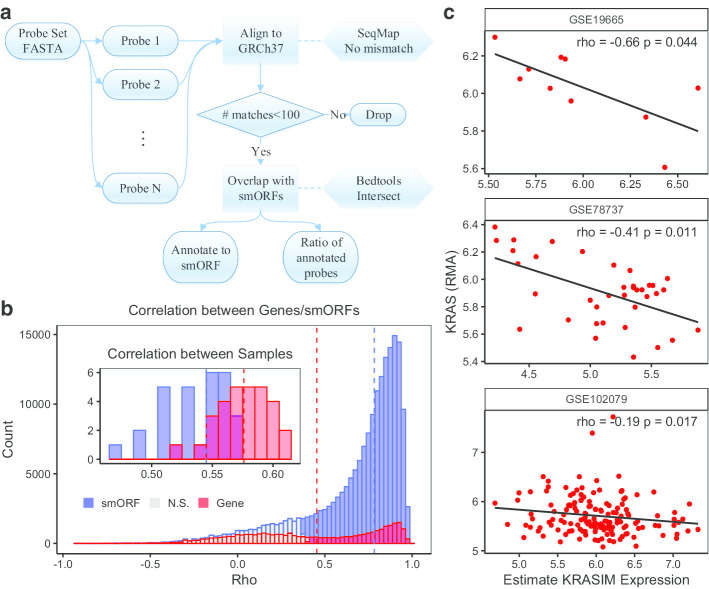
smORF RNA quantification based on microarray reannotation. **a** The workflow of reannotating microarray probes to smORFs. **b** The correlation of the estimated gene or smORF expressions between RNA sequencing and microarray. Spearman correlation were calculated between samples or between genes/smORFs and p values were adjusted using Benjamini–Hochberg procedure. Blue: smORF, red: gene, grey: not significant (FDR ≥ 0.05). **c** The Spearman correlation between KRAS and estimated KRASIM expression in 3 different datasets

Microarray is one of the most common transcriptome quantification methods especially before the invention of RNAseq. Although RNAseq is more sensitive than microarray and have less noises [20], microarray requires fewer computational resources and has generally well similarity with RNAseq [21]. Studies using microarrays, such as the IMI MARCAR Project [22] and Microarray Innovations in Leukemia (MILE) [23], have made great contributions to medical researches. We collected 617,462 unique smORFs from SmProt [8], sORFs.org [7] and the study by Thomas et al. [9]. Using probe reannotation, we remaped the probes of microarrays to smORFs and estimated smORF RNA expressions (Fig. 1a, Method).

Then we tested the accuracy of this quantification. By comparing smORFs and known RNAs (Ensembl v75) using the samples that underwent both RNAseq and microarray, the correlations between the samples decreased in smORFs, but the correlations between the RNAs increased (Fig. 1b). For example, KRASIM is a 99-aa microprotein expressed in hepatocellular carcinoma cells, whose overexpression reduces the level of KRAS [14]. In three datasets from Gene Expression Omnibus (GEO), KRASIM expression estimated by our method were significantly negatively correlated with expression of KRAS (Fig. 1c), which does not match the same probe as KRASIM, suggests that our method could effectively evaluate the expression of smORFs.

### Prediction of microprotein function based on expression similarity

Because of the large abundance of smORFs, it is difficult to construct a co-expression network like previous studies. Calculating correlations between smORFs and genes requires billions times of calculations, which is time-consuming and difficult to store and search. Inspired by the nearest neighbor algorithm, we built a BallTree for each dataset to find the nearest neighborhoods (genes) of smORFs. The estimated expressions of genes and smORFs in each dataset are converted to their rank orders by row (gene/smORF). We used Pearson correlation distance metric to measure the distances between nodes, which is equivalent to Spearman correlation since the expressions were converted to ranks in advance, but the time efficiency is greatly improved. By using the pre-ranking strategy and BallTree algorithm, the time consumption of searching correlated genes changed to 6% of that of no pre-ranking and brute force searching (Table 1).

**Table 1:** The time consumption of searching correlated genes using different methods

Time (s)	Brute force No pre-ranked	Brute force Pre-ranked	BallTree Pre-ranked
Pre-rank	–	0.344 (± 0.00299)	0.348 (± 0.00805)
Build model	–	–	21.7 (± 0.166)
Search	17.9 (± 0.157)	1.713 (± 0.0445)	1.08 (± 0.471)

The algorithms were run on Intel(R) Core(TM) i7-7700HQ CPU with 24 GB RAM

Using this speed-optimized correlation algorism, we calculated the Spearman correlation between smORFs and other known genes. Furthermore, the functions of smORFs/microproteins can be predicted using correlated genes through pathway enrichment analysis. Considering that biomolecules have different functions in different tissues and diseases, we collected microarray data from 48 tissues/cells and 82 diseases (and normal) involving 173 data sets and built prediction models respectively. Moreover, by aggregating the predictions of multiple models, we could get more reliable results. After applying our method to several microproteins that have been studied, we found that our method could successfully predict the functions of these microproteins.

**Fig. 2:**
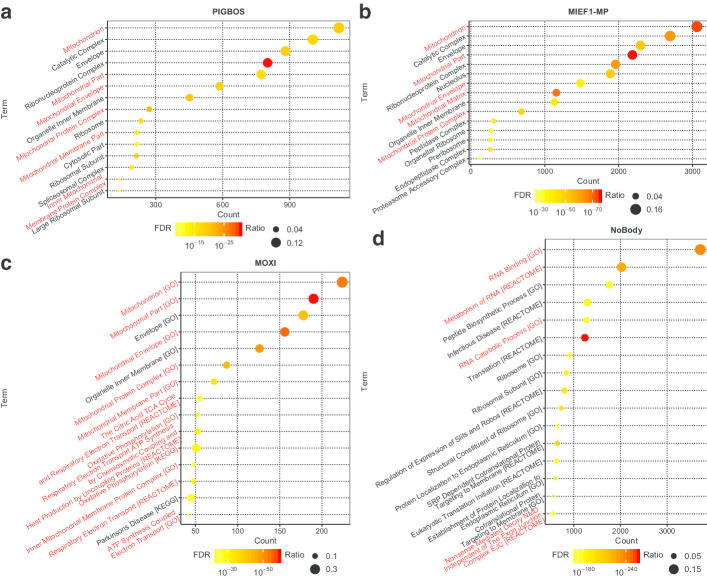
Function prediction of 4 known microproteins. **a**, **b** The predicted gene ontology cellular components of **a** PIGBOS and (b) MIEF1-MP. **c**, **d** The predicted function terms of **c** MOXI and **d** NoBody. Related terms were marked in red. FDRs were calculated using Benjamini–Hochberg procedure

For instance, phosphatidylinositol glycan anchor biosynthesis class B opposite strand 1 (PIGBOS), a 54-aa microprotein, as well as mitochondrial elongation factor 1 microprotein (MIEF1-MP), a 70-aa microprotein, were both located in mitochondrion [24, 25]. By merging the results of multiple datasets of normal tissues, our method successfully predicted their subcellular location in mitochondrion (Fig. 2a, b).

Additionally, micropeptide regulator of b-oxidation (MOXI), a 56-aa microprotein encoded by muscle-enriched long non-coding RNAs (lncRNA) LINC00116, was found to be located in mitochondrion and enhance fatty acid β-oxidation [15, 16]. By applying our method to several expression datasets of skeletal muscle tissues, we successfully predicted not only its cellular localization, but also the enrichment of cellular respiratory pathways such as oxidative phosphorylation (Fig. 2c).

Moreover, non-annotated P-body dissociating polypeptide (NoBody), translated from LOC550643, was previously found to interact with the mRNA decapping complex, which involves in RNA degradation and mediates nonsense mediated decay (NMD) [26]. Using our method in a variety of normal tissue datasets, the functions of NoBody in RNA metabolism and NMD were successfully predicted (Fig. 2d).

Lastly, mitochondrial micropeptide-47 (Mm47) is a 47-aa mitochondrial microprotein impacts the activation of the Nlrp3 inflammasome [17]. Although this microprotein is not annotated in the three studies we collected, the result of basic local alignment search tool (BLAST) [27] shows its high similarity to a 21-aa microprotein located at chromosome 7 (+):135358848–135358913 (GRCh37) (Additional file 1: Figure S1a). It is reasonable to consider that they have similar functions. Prediction of the function of this 21-aa microprotein in normal tissues shows that it was located in mitochondrion, which is the same as Mm47 (Additional file 1: Figure S1b).

### Further validation of prediction process

**Fig. 3:**
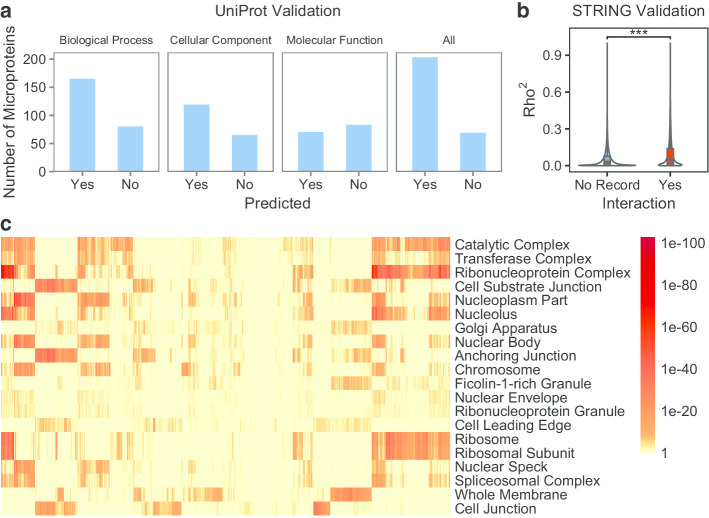
Further validation of smORFunction and the overview of microprotein cellular component. **a** The number of microproteins whose at least one gene ontology function was successfully predicted or not. **b** The Spearman’s correlation coefficients (Rho^2^) of microprotein–protein pairs which have interaction records in STRING database or not. **c** The FDRs of predicted gene ontology cellular components of random 10,000 microproteins. Top 20 terms with the most number of significant (FDR < 0.2) results were shown

To further observe the validity of our approach, we collected 270 microproteins from the Universal Protein Resource (UniProt) [28], as well as corresponding GO functional annotations. Using the Genotype-Tissue Expression (GTEx) microarray data set (GSE45878), we predicted the functions of these microproteins. The results showed that at least one function of 202 microproteins (74.8%) could be successfully predicted (Fig. 3a). Moreover, we downloaded the human protein interactions from the STRING [29] database. Only interactions involving the microproteins we collected were retained. Using the estimated microprotein RNA expression from the GTEx microarray dataset, we calculated the expression correlation between microprotein RNA and known genes. We found that the correlation coefficients (Rho^2^ of Spearman’s test) of the microprotein-protein pairs with the interactions were significantly higher than those of the pairs without interaction records (Fig. 3b). These results further demonstrate the accuracy of our method for the quantitative measurement and functional prediction of smORFs.

### The cellular component overview of microprotein

Using our method, we explored the cellular components of microproteins. First, we randomly selected 10,000 microproteins. Then we selected up to 1000 positively related known genes for each microprotein using the GTEx microarray dataset. The cellular components of these microproteins was predicted by enrichment analysis. The results showed that 52.04% of the microproteins were predicted to be associated with the catalytic complex (FDR < 0.2, Fig. 3c). The first ranking of the catalytic complex did not change when a stricter FDR (FDR < 0.05) was used. Followed by transferase complex and ribonucleoprotein complex, with 44.95% and 44.90%, respectively. The possible reason is that the relatively large size of these gene sets (1355, 778, and 680) makes it more possibly to have significant results. On the other hand it also means that unknown proteins are more likely to belong to these components, providing a potential direction for future research.

### A web tool for microprotein function prediction

**Fig. 4:**
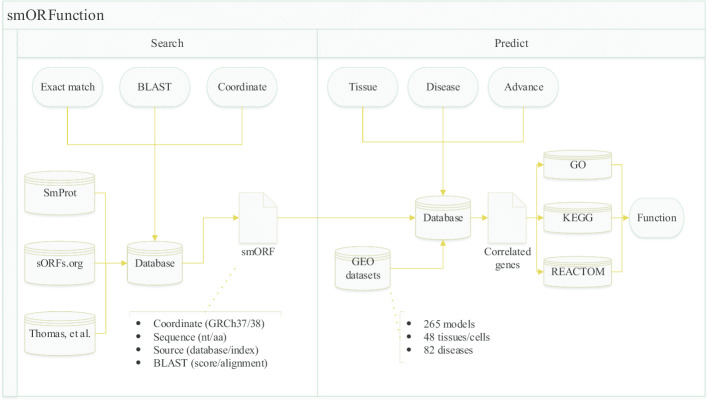
The workflow of our smORF/microprotein function prediction web tool, smORFunction. The tool is online at https://www.cuilab.cn/smorfunction

By the advantage of the speed-optimized correlation algorism, it is possible to perform prediction while requesting. We developed our method into a web tool, smORFunction (https://www.cuilab.cn/smorfunction), which contains 617,462 unique smORFs annotated by SmProt, sORFs.org and the study of Thomas et al. smORFs can be searched by sequence using exact mode or BLAST, or by coordinate in reference genome (GRCh37 or GRCh38). For 526,443 smORFs that can be mapped to at least one probe of one microarray platform, we provide functional predictions in at most 48 tissues/cells, 82 diseases (and normal), including GO terms, KEGG pathways, and REACTOM pathways (Fig. 4). This tool will provide inspirations for the research on the functions of smORFs and microproteins.

## Discussion

Similarities based on networks are widely used to predict the functions of proteins and non-coding RNAs [30]. Using protein–protein interaction network, the functions of unknown proteins can be annotated by interacted proteins with known functions [31, 32]. The functions of microRNAs (miRNAs) can be predicted based on upstream transcription factor regulation network [33] or downstream target gene network [34]. Non-coding RNA function annotation server (ncFANs) used coding-non-coding gene co-expression network to annotate lncRNA functions [35].

Some of the existing smORF/microprotein function prediction tools also used the network for function prediction. ProteomeHD used MS to identify the co-regulation of proteins and to predict the functions of proteins and microproteins [18]. FSPP annotated the functions of microproteins through co-expression and co-location networks constructed by MS, Ribo-seq and RNAseq [19]. The quantification of smORFs using RNAseq or MS requires more computational resources and time. Given the small molecular weight of microproteins, only a few microproteins can be detected and quantified by MS. In contrast, microarrays allow faster access to more smORFs of more datasets. ProteomeHD covered ~ 10,000 proteins, a small fraction of which were microproteins, much smaller than the potential number of microproteins, while FSPP used 38 samples from 5 human cell lines. In our research, prediction models for up to 526,443 smORFs are provided, involving 48 tissues/cells, 82 diseases (and normal).

Additionally, the consistency of microarray quantification of smORFs with RNAseq is similar to that of known genes, suggests that our method could effectively evaluate the expression of smORFs. But there is a phenomenon that the correlations between the samples decreased in smORFs, but the correlations between the RNAs increased. We think this may be due to the large number of smORFs. There are about 20,000 known genes, whereas we quantified about 500,000 smORFs. This makes it more likely that the smORFs contain outliers that make correlations decrease. But for calculating the correlations between RNAs, smORFs and known genes use the arrays of the same length (number of samples). We think this is more comparable. Moreover, when calculating correlated genes, we only focus on the correlations between RNAs, making this measurement more important than the correlations between samples. Our quantification of smORFs obtained higher correlations between RNAs than known genes, suggesting that our re-annotation and quantification process is reliable enough.

Building networks for hundreds of thousands of smORFs is difficult, so we simplified this step using Spearman’s correlation, equating to building a two-layer smORF-gene network. Based on the reannotation of microarray probes, our tool predicts the function of smORFs by correlated genes with functional annotation. Although protein and RNA are often inconsistent [36, 37], and it is difficult for microarray to evaluate the expression of transcripts with low abundance and those without intersection with the probes [20], our method still achieved well prediction performance. Furthermore, our tool includes more smORFs and more models of different tissues and diseases than existing tools.

Microarray platforms usually have tens of thousands of probe sets, but are still far fewer than potential smORFs. 526,443 of all the smORFs we collected can be annotated by at least one probe set of one platform. Although RNAseq can be used to evaluate the expression levels of all the smORFs, the process of sequence alignment and counting reads requires more time and computational resources. Meanwhile, MS quantification also requires massive calculations, and not all microproteins can be detected. In addition, the same probe may match multiple genes and/or smORFs, resulting in inaccurate estimation of the expression of smORFs. This non-unique mapping problem also exists in RNAseq and MS. Research shows that similar sequences may have similar functions [38]. Other study shows that near transcripts in the genome tend to have similar functions [39]. Therefore, it is reasonable to think that the smORFs that match to the same probe may have similar functions. Besides, these genes and smORFs share the signal intensity of the same probe in unknown proportions. We hypothesize that these proportions remain consistent across samples from the same dataset, tissue, and disease. Based on this assumption, it can be calculated that regardless of these unknown proportions, the Spearman’s correlation between smORFs and other genes is constant, so the predictions remain unchanged, reducing the impact of quantitative inaccuracies caused by non-unique mapping.

## Conclusions

In summary, we collected 617,462 unique smORFs from SmProt, sORFs.org, and the study of Thomas et al. By reannotating the microarray probes, 526,443 smORFs are matched to the probes. The expression of smORFs was estimated by these rematched probes, and the accuracy of this quantitative method was evaluated. Furthermore, we collected 173 datasets from the GEO, including 48 tissues/cells, 82 diseases (and normal) and generated 265 prediction models. The functions of the smORFs were predicted by correlation analysis and pathway enrichment. After applying our method to 270 known microproteins from literatures and database, our method generally performs well. Finally, we developed our method into a web tool, smORFunction, which could provide references for the functional researches of smORFs and microproteins.

## Methods

### The collection of smORFs

The annotations of smORFs were accessed from SmProt (https://bioinfo.ibp.ac.cn/SmProt/) [8], sORFs.org (https://sorfs.org/) [7], and the study of Thomas et al. [9]. The coordinate information of the three databases is GRCh37, GRCh38 and GRCh37, respectively. CrossMap (v0.3.0) [40] was used to map the coordinate of smORFs to the other reference genome, respectively. The same internal IDs were given to the same smORFs. 617,462 different human smORFs were eventually collected. The Gene Ontology (GO) terms of known microproteins were collected from the Universal Protein Resource (UniProt, https://www.uniprot.org/) [28]. The human protein–protein interactions were obtained from STRING database (https://string-db.org/) [29].

### Omics data collection

The raw files for RNAseq (SRA) and microarray (CEL) were downloaded from the GEO datasets. GSE104610 and GSE104973 respectively used microarray and RNAseq to conduct RNA quantification on samples that had undergone the same treatment, which was used to evaluate the accuracy of probe reannotation in our study. In addition, 173 microarray data of disease and/or normal tissue samples were collected for functional prediction of smORFs.

### Microarray data processing

The CEL files were processed using R package oligo (v1.48.0) [41] and ff (v2.2-14). Package ff was used with default parameters. Samples from different datasets were separated for background correction and normalization, and the probe signals were estimated using Robust Multichip Average (RMA) algorithm. The probes were annotated to Entrez IDs by Ensembl BioMart. The duplicate Entrez IDs were aggregated by their median.

### RNAseq data processing

SRA files were converted into fastq files by SRA Toolkit (v2.9.6), and quality controls were carried out by fastp (v0.20.0) [42]. We used HISAT2 (v2.1.0) [43] to align sequences in fastq files to the reference genome GRCh37, using default parameters. SAM files are converted to BAM files using SAMtools (v1.9) [44] and sorted. featureCounts (v2.0.0) [45] was used to count reads to coding and non-coding RNAs (Ensembl v75) and smORFs we collected. Parameters '–p –t exon –g gene_id' were used for the quantification of Ensembl RNAs. Given that there are many overlapping smORFs, the -O parameter is additionally used when counting reads of smORFs. Read counts were finally normalized as fragments per kilo-base per million mapped reads (FPKM).

### Probe reannotation

Affymetrix microarray (HTA 2.0, hg u133 plus 2, hg u133a, hg u133b, HuGene 1.0 st v1, HuGene 2.0 st v1, HuEx 1.0 st v2) probe sequences were downloaded from the website of Affymetrix (https://www.affymetrix.com/support/technical/byproduct.affx). Each probe set contains several different probes. We used SeqMap [46] to align the probe sequences to GRCh37, using /output_all_matches and /do_not_output_probe_without_match parameter and number of mismatches was set to 0. Probes that can be 100% matched and have fewer than 100 matches are retained, the same parameters as which were used in BioMart (https://www.ensembl.org/info/genome/microarray_probe_set_mapping.html). Next, the probes' coordinates are intersected with those of smORFs, using BEDTools (v2.26.0) [47]. The probe sets with at least one base intersection is annotated to the corresponding smORF. At the same time, the proportion of the probes that overlap with such smORF in the probe set to all the remaining probes in the probe set is also calculated.

### Estimation of smORF expressions

Totally *n* different probe sets are annotated to a smORF with proportions (weights) *w*_*1*_, *w*_*2*_, …, *w*_*n*_. The probes with *weight ≤ 0.1* were removed. Given that RMA normalization takes the signal intensities to the logarithm, we used the exponentiation to reverse this process. By multiplying the *weight* with the signal intensity, we obtained the estimated smORF expression. For the same smORFs that could match multiple probes. we evaluated the median, mean, and maximum expressions by comparing the performs of ‘correlations between RNAs’, and finally chose the median expression. The signal strength of these probes in the sample is *RMA*_*1*_, *RMA*_*2*_, …, *RMA*_*n*_. Then the expression *E* of this smORF is estimated as:

$${\text{E}} = \log_{2} {\text{median}}\left( {w_{1} 2^{{RMA_{1} }} ,w_{2} 2^{{RMA_{2} }} , \ldots , w_{n} 2^{{RMA_{n} }} } \right) (w_{i} > 0.1)$$

### Finding correlated genes

Spearman correlation was used to calculate the correlation between smORF and known genes (Entrez ID). The expressions of genes and smORFs in each dataset are converted to their rank orders by row (by gene/smORF). The records that have the same value were ranked using their mean rank. We built a BallTree for each dataset to find the nearest neighborhoods (genes) of smORFs. The leaf size was set as 5 after trying different parameters to find the one with the best time efficiency. We used Pearson correlation distance metric to measure the distances between nodes:

$${\text{Distance}} = 1 - \frac{{cov\left( {rank_{gene} ,rank_{smORF} } \right)}}{{\sigma_{{rank_{gene} }} \cdot \sigma_{{rank_{smORF} }} }}$$

where rank_gene_ and rank_smORF_ are the ranks of the expressions of a gene and a smORF in a dataset, respectively. This is equivalent to Spearman correlation since the expressions were converted to ranks in advance, but the time efficiency is greatly improved.

### Function prediction

By default, in dataset S, at most 1000 genes with rho ≥ 0.5 were retained, and the number of these genes is *N*_*S*_. We obtained the functional annotated gene sets of GO [48, 49] (including biological process, cellular component, and molecular function), Kyoto Encyclopedia of Genes and Genomes (KEGG) [50] and REACTOM [51] from Molecular Signatures Database (MSigDB, https://www.gsea-msigdb.org/gsea/msigdb) [52]. Hypergeometric distributions are used for evaluate function prediction. The total background genes *T*_*S*_ were set to be the intersection of the gene that can be annotated by the probes with all genes contained in all functional gene sets. For the functional gene set containing *M*_*S*_ genes, *I*_*S*_ genes were screened to be correlated. Then p value can be calculated as follows:

$${\text{p}}_{S} = \mathop \sum \limits_{{x = I_{S} }}^{{N_{S} }} \frac{{\left( {\begin{array}{*{20}c} {M_{S} } \\ x \\ \end{array} } \right)\left( {\begin{array}{*{20}c} {T_{S} - M_{S} } \\ {N_{S} - x} \\ \end{array} } \right)}}{{\left( {\begin{array}{*{20}c} {T_{S} } \\ {N_{S} } \\ \end{array} } \right)}}$$

For all datasets, a summarized *p* value can be calculated:

$${\text{p}} = \mathop \sum \limits_{{x = \sum I_{S} }}^{{\sum N_{S} }} \frac{{\left( {\begin{array}{*{20}c} {\sum M_{S} } \\ x \\ \end{array} } \right)\left( {\begin{array}{*{20}c} {\sum T_{S} - \sum M_{S} } \\ {\sum N_{S} - x} \\ \end{array} } \right)}}{{\left( {\begin{array}{*{20}c} {\sum T_{S} } \\ {\sum N_{S} } \\ \end{array} } \right)}}$$

Further, the false discovery rate (FDR) is estimated using Benjamini–Hochberg method. By default, functional terms with *p* ≤ 0.05 and FDR ≤ 0.2 are selected as the predicted functions of smORF.

### Statistical analysis

Wilcoxon rank sum tests were used to calculate the significance of difference between two groups of continuous variables. Correlation between two continuous variables were estimated using Spearman’s tests.

## Supplementary information

**Additional file 1: Figure S1.** The function prediction of Mm47 using its similar microprotein. (a) The alignment between Mm47 and smORF at chr7: 135358848–135358913 (+) using BLAST protein (BLASTp). (b) The prediction of gene ontology cellular components of the similar microprotein. Related terms were marked in red. FDRs were calculated using Benjamini–Hochberg procedure.

## Data Availability

The datasets generated during the current study are available in smORFunction website, https://www.cuilab.cn/smorfunction/download. The datasets used and analyzed during the current study are available from: (1) SmProt, https://bioinfo.ibp.ac.cn/SmProt/download.htm. (2) sORFs.org, https://sorfs.org/BioMart. (3) The study of Thomas, et al., https://static-content.springer.com/esm/art%3A10.1038%2Fs41589-019-0425-0/MediaObjects/41589_2019_425_MOESM3_ESM.xlsx. (4) STRING, https://string-db.org/cgi/download.pl. (5) Ensembl, https://asia.ensembl.org/info/data/ftp/index.html. (6) MSigDB, https://www.gsea-msigdb.org/gsea/downloads.jsp.

## Abbreviations

ORFs: Open reading frame
smORF: Small open reading frame
Ribo-seq: Ribosome profiling sequencing
MS: Mass spectrometry
FSPP: Functional smORF-encoded peptides predictor
MILE: Microarray innovations in leukemia
GEO: Gene expression omnibus
BLAST: Basic local alignment search tool
UniProt: Universal protein resource
GTEx: Genotype-tissue expression
GO: Gene ontology
KEGG: Kyoto encyclopedia of genes and genomes
