# Supplementary material for: smORFunction: a tool for predicting functions of small open reading frames and microproteins
Source: BMC Bioinformatics. 2020 Oct 14;21:455. doi: 10.1186/s12859-020-03805-x (PMC7559452; doi:10.1186/s12859-020-03805-x)
Supplement: Supplementary file 1 — Additional file 1: Figure S1. The function prediction of Mm47 using its similar microprotein. (a) The alignment between Mm47 and smORF at chr7: 135358848–135358913 (+) using BLAST protein (BLASTp). (b) The prediction of gene ontology cellular components of the similar microprotein. Related terms were marked in red. FDRs were calculated using Benjamini–Hochberg procedure. [file 12859_2020_3805_MOESM1_ESM.pdf]

a

```
BLAST Alignment Bit Score=38, Expectation=5.1e-6
                                     1                    21
smORF:chr7:135358848-135358913(+):  LAKKLEEIKKDLDAKKKPPSA
                                     LAKKLEEIKKDL+AKKKPPS+
 Mm47:   MLQFLLGFTLGNVVGMYLAQNYEMPNLAKKLEEIKKDLEAKKKPPSS
                                     27                   47
```

b

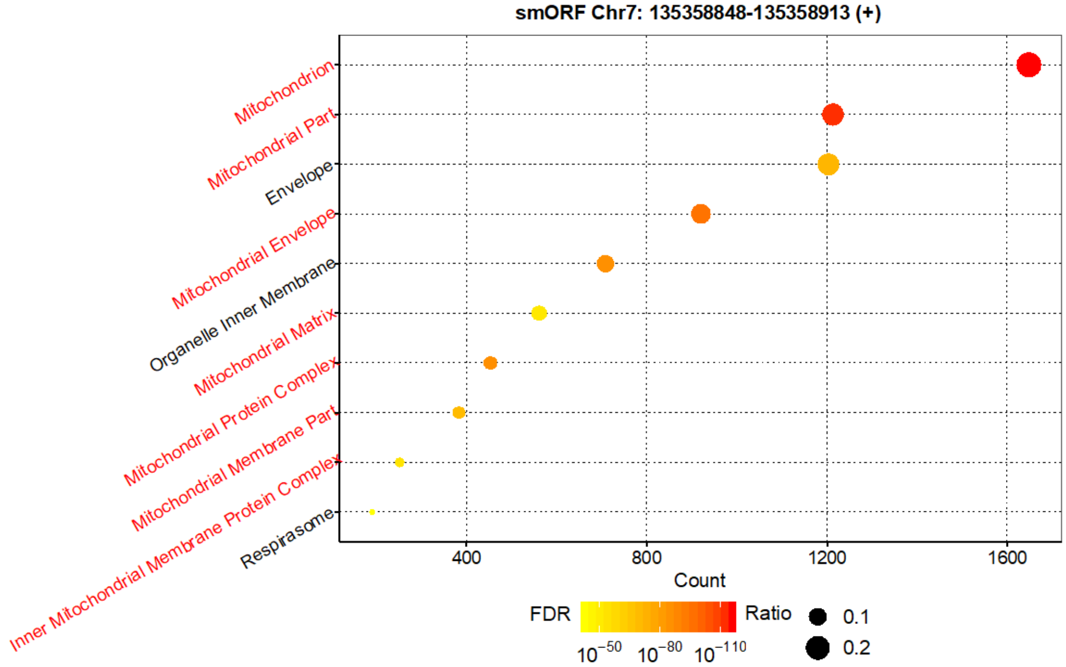

Supplementary Figure S1. The function prediction of Mm47 using its similar microprotein. (a) The alignment between Mm47 and smORF at chr7: 135358848-135358913 (+) using BLAST protein (BLASTp). (b) The prediction of gene ontology cellular components of the similar microprotein. Related terms were marked in red. FDRs were calculated using Benjamini-Hochberg procedure.
